# Best Practices for Evaluation and Treatment of Agitated Children and Adolescents (BETA) in the Emergency Department: Consensus Statement of the American Association for Emergency Psychiatry

**DOI:** 10.5811/westjem.2019.1.41344

**Published:** 2019-02-19

**Authors:** Ruth Gerson, Nasuh Malas, Vera Feuer, Gabrielle H. Silver, Raghuram Prasad, Megan M. Mroczkowski

**Affiliations:** * Bellevue Hospital/New York University, Department of Psychiatry, New York, New York; † University of Michigan, Departments of Psychiatry and Pediatrics, Ann Arbor, Michigan; ‡ Northwell Health, Department of Psychiatry, New Hyde Park, New York; § Weill Cornell Medical College, Department of Psychiatry, New York, New York; ¶ Children’s Hospital of Philadelphia, Department of Psychiatry, Philadelphia, Pennsylvania; || Columbia University Medical Center, Department of Psychiatry, New York, New York; * Bellevue Hospital/New York University; † University of Michigan; ‡ Northwell Health; § Weill Cornell Medical College; ¶ Children’s Hospital of Philadelphia/University of Pennsylvania School of Medicine; || Columbia University Medical Center; # Kings County Hospital Center; ** Tufts University School of Medicine; †† Brown University; ‡‡ University of California, Los Angeles; §§ Hartford Hospital/Institute of Living/University of Connecticut School of Medicine; ¶¶ University of California, San Diego; |||| Massachusetts General Hospital/Harvard Medical School

## Abstract

**Introduction:**

Agitation in children and adolescents in the emergency department (ED) can be dangerous and distressing for patients, family and staff. We present consensus guidelines for management of agitation among pediatric patients in the ED, including non-pharmacologic methods and the use of immediate and as-needed medications.

**Methods:**

Using the Delphi method of consensus, a workgroup comprised of 17 experts in emergency child and adolescent psychiatry and psychopharmacology from the the American Association for Emergency Psychiatry and the American Academy of Child and Adolescent Psychiatry Emergency Child Psychiatry Committee sought to create consensus guidelines for the management of acute agitation in children and adolescents in the ED.

**Results:**

Consensus found that there should be a multimodal approach to managing agitation in the ED, and that etiology of agitation should drive choice of treatment. We describe general and specific recommendations for medication use.

**Conclusion:**

These guidelines describing child and adolescent psychiatry expert consensus for the management of agitation in the ED may be of use to pediatricians and emergency physicians who are without immediate access to psychiatry consultation.

## INTRODUCTION

### Background

Agitation and aggression in children and adolescents in the emergency department (ED) can be dangerous and distressing for patients, families and staff.^1^ Agitation and aggression can disrupt care, cause injury, or necessitate use of physical restraint. Of youth presenting to the ED for psychiatric care, 6–10% require restraint.^2^–^3^ At least 30 children in the United States (U.S.) have died in restraint-related incidents, which has led to regulations limiting the use of restraint to emergencies where least restrictive options have been exhausted.^4^–^5^ There is little guidance or standardization toward use of less restrictive options, especially medications, to manage agitation and avoid restraint.

There are no randomized controlled trials, expert consensus guidelines, or comparative studies of medication efficacy or safety in the ED setting. A survey of emergency physicians (EP) regarding pro re nata (as needed) (hereafter referred to as STAT/PRN) medications commonly used for agitation, and review papers providing recommendations for medication use, all emphasize use of first- and second-generation neuroleptics, benzodiazepines, and mood stabilizers.^2^,^6^–^9^ These are largely inspired by consensus guidelines for treatment of agitated adults or pediatric outpatients with chronic aggression.^10^–^12^ Symptoms and triggers that underlie agitation in the ED may be different from those that underlie chronic aggression among outpatients.^13^

A small number of studies have examined the use of STAT/PRN medications for acute agitation in psychiatrically hospitalized youth. There is only one randomized, placebo-controlled study of STAT/PRN medication for acute agitation, which found no difference between diphenhydramine vs placebo.^14^ Intramuscular (IM) administration (of either diphenhydramine or placebo) was significantly more effective than by mouth (PO) administration. A retrospective study of STAT/PRN medications in 49 psychiatrically hospitalized youth reported antihistamines were used most commonly, followed by neuroleptics and sedative-hypnotics.^15^ Only 32% of all PRNs were clearly effective on chart review. Benzodiazepines and neuroleptics were equally efficacious, and IM administration was significantly more effective than PO administration across medication classes.

A retrospective study of STAT/PRN medications among psychiatrically hospitalized youth found that olanzapine was more likely than lorazepam or chlorpromazine to produce a “settling effect” within 30 minutes or less; all were generally well tolerated, although the authors noted that a small number of youth experienced paradoxical agitation with lorazepam.^16^ Two case-controlled, retrospective, chart-review studies have assessed the relative efficacy of IM ziprasidone, compared to other IM neuroleptics, in psychiatrically hospitalized adolescents. The first compared IM ziprasidone to IM olanzapine; there was no significant difference in efficacy, although ziprasidone subjects received significantly more emergency medications.^17^ A second compared the combination of IM haloperidol with IM lorazepam and IM ziprasidone. There was no significant difference found in restraint duration, use of STAT/PRN medications, or vital sign changes.^18^

### Importance

These studies have limited generalizability to STAT/PRN use of these medications for acute agitation or aggression in ED settings. Without evidence-based or expert consensus guidelines to direct decision-making, physicians in the ED setting typically use medications with which they are most comfortable, although these may not be the most effective or safest choice with significant variance in practice.^2^,^6^

**Population Health Research Capsule**

What do we already know about this issue?

Pediatric agitation in the emergency department (ED) is both prevalent and challenging with no existing standard, despite the need for careful multidisciplinary evaluation and management.

What was the research question?

Can an evidence-based, consensus guideline be developed for the management of pediatric agitation in the ED?

What was the major finding of the study?

Evidence-based, expert consensus guidelines for management were developed including etiology-driven treatment strategies.

How does this improve population health?

Standardizing pediatric agitation management in the ED supports consistent and evidence-based care for patients and staff at risk for injury and negative outcomes.

### Goals of Investigation

We aim to present consensus guidelines for management of agitation among pediatric patients in the ED, including use of STAT (for immediate administration) or STAT/PRN medications, in follow up to the Consensus Statement of the American Association for Emergency Psychiatry (AAEP) Project BETA Psychopharmacology Workgroup guidelines for agitation in adults.^10^

## METHODS

### Study Design and Setting

Given the dearth of child psychiatrists in the U.S., this workgroup focused on the consensus of a group of experts in this subspecialty. The workgroup was assembled from experts in emergency child and adolescent psychiatry and psychopharmacology from the AAEP, the American Academy of Child and Adolescent Psychiatry (AACAP) Emergency Child Psychiatry Committee, and peer recommendation. Sixteen experts participated, all board certified in child and adolescent psychiatry with some additionally board certified in pediatrics. The experts represented 14 hospitals in eight states.

### Interventions

The non-voting project chair (RG) facilitated discussion, information gathering, and consensus building. Consensus was obtained using consensus development methodology, specifically the Delphi method, which was developed to obtain reliable opinion consensus and avoid bias.^19^–^20^ Per the Delphi method, opinions were elicited from the experts through a series of emailed questionnaires and structured solicitation of feedback. There were six rounds of questionnaires and feedback in total, starting with determining the structure of the guidelines (by age/weight, medication class, severity or etiology of agitation), and then narrowing progressively to choose the assessment strategies, etiologic categories, medications, doses, and cautions noted below. In the first of these rounds of questionnaires, experts assessed the standardized review of the existing literature on management of agitation summarized above, as well as published and unpublished guidelines and protocols used by EDs across the country (solicited through AAEP, AACAP, and outreach to several EDs and experts in the field). All opinions were anonymized and aggregated by the project chair to avoid direct confrontation between experts and prevent bias. This manuscript also underwent two rounds of workgroup feedback.

## RESULTS

The following summarizes the consensus recommendations for the evaluation and pharmacological management of agitation among pediatric patients in the ED.

### Multimodal Approach

There is consensus that management of agitation in the ED should be individualized, multidisciplinary, and collaborative. Medication should serve as one part of a comprehensive strategy to address the behavior. Clinicians should attempt to understand the etiologic factors leading to agitation, use non-pharmacologic de-escalation strategies, and choose medication based on the patient’s specific needs and history. For example, consider a child with autism who is brought to the ED for aggression triggered by anxiety, who then becomes agitated and attempts to flee the ED due to hunger and sensitivity to fluorescent lights. Effective treatment requires addressing his anxiety (considering non-pharmacologic and pharmacologic interventions), hunger, and sensory needs. In many cases, addressing etiologic factors proactively and non-pharmacologically can obviate or completely eliminate the need for pharmacologic management.

### Etiology Drives Choice of Treatment

There is consensus that, whenever possible, the etiology of agitation should be ascertained and all treatments targeted to the root causes of the agitation. Diagnostic assessment occurs in parallel with symptomatic management. Collateral information, response to non-pharmacologic interventions, mental status and change in symptoms over time inform this ongoing assessment. While standardized scales are often used in adult settings, there are few broadly used, evidence-based tools for pediatric agitation; thus, thoughtful clinical assessment is imperative. Cross-disciplinary collaboration and communication is also key to identifying potential causes of agitation. The bedside nurse is uniquely suited to notice changes in the patient’s mental status or behavior, implement non-pharmacologic strategies early, and quickly engage crisis services. Family members provide a crucial premorbid developmental and behavioral baseline of their child and may help elucidate the cause of agitation.

The assessment of etiology starts with asking why the child has become agitated now and here, considering antecedents such as environmental or interpersonal triggers, as well as internal stressors such as pain or acute psychiatric symptoms. Psychiatric history, medication review, including any potential for toxic ingestion (intentional or accidental), allergies, past medical history, developmental history and a focused social and family history, including trauma history, should also be obtained.^21^

Medical evaluation of agitated patients is critical, although completing a full physical examination and any indicated laboratory/imaging studies may be challenging during acute agitation. If the etiology of agitation is unknown or mixed, there is consensus that the clinician should use best clinical judgment and provide symptomatic management based on available diagnostic and clinical information. The clinician should continuously reevaluate the differential diagnosis, observing response to intervention closely, and adjust diagnostic assessment and management accordingly.

### Differential Diagnosis

Agitation is a symptom, like pain, with many potential etiologies and often multiple factors contributing in the moment. The potential etiologies for acute agitation among youth in the ED includes physical disease (such as pain, delirium, intoxication and catatonia), anxiety, developmental and cognitive disabilities, behavioral disorders, trauma, mania, psychosis, sensory or physical limitations, and difficulty communicating needs. Even if a child has a known history of psychiatric or developmental disorders, comorbid physical disease, anxiety or other acute triggers should still be ruled out and a broad differential maintained. Non-pharmacologic approaches used for de-escalation should be employed early with a preventative, proactive approach.

### Non-pharmacologic Management

There is consensus that non-pharmacologic approaches should be used to prevent and de-escalate agitation before pharmacologic measures are considered. A multidisciplinary approach allows primary and secondary prevention strategies. Primary prevention includes changes to the ED environment to make youth more comfortable, clear communication to reduce anxiety, and effective assessment and treatment of pain and other acute physical symptoms. Secondary prevention includes modifications for youth identified to be at baseline elevated risk for agitation or for youth beginning to show signs of agitation. Family members may identify calming strategies that have been effective in the past, which may contribute to crisis and behavioral planning. An agitated child should be moved away from other patients to a calming, safe area without access to sharps and dangerous objects.^21^

Even if a youth in the ED is becoming highly agitated, simple non-pharmacologic de-escalation strategies can be effective and should always be attempted before, with, and after pharmacotherapy. Communicating in a neutral yet empathic tone, communicating at the patient’s eye level, and using clear, concrete and simple language (or visual communication tools for youth with developmental disabilities) are helpful. Reunification with (or separation from) family members, food, drink, distraction, preferred comfort items from home, or sensory coping kits can ease tension. Firm limits on unacceptable behaviors and specific praise for adherence to requests and de-escalation mold behavior while also modeling for families how to parent in the face of disruptive behaviors. Reflective statements and validation help youth who struggle with articulating complex emotions feel understood, while clarifying triggers for agitation and promoting problem-solving.

### Rationale for Medication Use

The goal of pharmacotherapy is twofold: 1) target the underlying cause of distress; and 2) calm the patient sufficiently for rapid assessment and treatment.

While medication for agitation is often considered when non-pharmacologic interventions have “failed,” pharmacologic and non-pharmacologic strategies should be used in concert with non-pharmacologic de-escalation efforts continuing during and after medication administration. When medication is used, it should be calming but not excessively sedating, as a youth who is asleep cannot be evaluated, participate in care, or leave the ED. Medication should be chosen for its calming effect but also to address the underlying etiology of the youth’s distress, so no one medication will be appropriate for all patients or all types of agitation.

### General Recommendations Regarding Medication Use (Table 1, Table 2)

A current medication list and medication history (including prior STAT/PRN medication use) helps to avoid drug interactions and adverse drug events (ADEs) and inform medication choice and dosing. Often a half dose or extra dose of a home medication can ameliorate escalating agitation. There is neither firm evidence nor consensus to support the use of one medication or even class of medication for all patients. Risks of ADEs should be weighed against potential benefit, while considering patient age, weight, medical comorbidity, and development when choosing a medication. There is consensus that PO administration should be tried whenever possible before the IM route. If intravenous access is already in place and safely accessible, this is preferred to IM administration. Neuroleptics should be used judiciously, only when truly indicated, and with appropriate monitoring, given potential adverse effects, particularly extrapyramidal adverse effects. Response to any intervention should be observed and documented closely.

Diphenhydramine, benzodiazepines, and alpha-2 agonists are generally calming and can also provide symptom-focused treatment. Diphenhydramine, with a more benign ADE profile and greater familiarity among families and medical providers, should be considered for younger children, youth with mild to moderate anxiety, youth with severe anxiety not secondary to delirium, intoxication, or withdrawal, and youth with mild agitation and no clear psychiatric or significant physical health history. Diphenhydramine and benzodiazepines should be avoided in delirium or in children where there is history of, or concern for, paradoxical disinhibition. Alpha-2 agonists can also provide symptomatic management of anxiety, hyperactivity, and hyperarousal, although these medications require blood pressure monitoring.^22^

Neuroleptics can be considered for most causes of severe agitation. Total daily dose should be monitored closely. Olanzapine can potentially be more sedating than haloperidol or risperidone and has less risk for cardiac adverse events or extrapyramidal symptoms. Given the risk of respiratory suppression if given concomitantly with benzodiazepines, olanzapine and benzodiazepines should not be administered parenterally within one hour of each other.^23^ Despite the studies noted above of PRN ziprasidone for agitation in psychiatric inpatients, there is consensus that ziprasidone is not recommended due to its activating potential, QT prolongation risk, and need for concomitant food intake when administered PO.

There is consensus that if an initial dose of medication was ineffective, a second dose of the same medication is preferable to adding multiple different medications (unless limited by ADE), as children can be vulnerable to drug-interaction adverse effects. An exception to this was combining haloperidol and lorazepam, which was generally considered preferable to a second dose of a neuroleptic in non-delirious patients. The etiology of agitation should be reassessed continuously, especially after two doses of a particular medication, and youth who have received multiple doses should be monitored continuously. Total daily dose or not to exceed instructions should be written and cumulative doses monitored, lest akathisia, delirium, and iatrogenic syndromes such as neuroleptic malignant syndrome be misperceived as worsening agitation.

There is consensus that ketamine and barbiturates are not recommended for treatment of agitation and that opioid analgesics should not be used for agitation unless for pain control.

### Specific Guidelines for Medication Use (Figure)

Below are the consensus medication regimens for the five most common etiologies of agitation: delirium; substance intoxication/withdrawal; developmental disability-related; psychiatric diagnosis; and unknown ctiology. Youth may present with agitation of mixed etiology, for example an adolescent with bipolar disorder who presents intoxicated, or a child with autism spectrum disorder who is delirious secondary to medical illness. In such complex cases, the ED clinician should use his or her best judgment in assessing the relative contribution of each etiologic factor to the presentation and strongly consider consulting child and adolescent psychiatry or other pediatric subspecialists for assistance.

#### Agitation Due to Delirium

Delirium is a complex clinical syndrome in which underlying physical disease, pharmacologic factors or both cause acute onset of mental status change with fluctuating course, involving symptoms of inattention, altered level of awareness and other cognitive deficits.^24^ Management of delirium requires identification and treatment of underlying etiologies. The initial approach should include reduction or discontinuation of medications that may be causing or exacerbating delirium. Pain should be treated while avoiding over-sedation and limiting exposure to opioid analgesia, which can worsen delirium. Medications may be needed to address underlying etiologies potentiating delirium, support sleep, and ameliorate physical symptoms such as pain or nausea. Medication for agitation can be necessary for safety, as well as avoiding medications that may worsen confusion or behavior in the setting of delirium, namely anticholinergics, benzodiazepines, and opioid analgesics.

Neuroleptics are the most commonly used pharmacologic intervention for delirium. Second-generation neuroleptics such as risperidone, olanzapine, and quetiapine have eclipsed haloperidol as the first-line agents.^25^–^27^ Choice of neuroleptic should account for the patient’s particular needs including route of administration, time to effect, potential side effects, illness factors, patient past experience with neuroleptics, and the specific symptoms of delirium being targeted. Clonidine may be used if there is reason to avoid neuroleptics. Melatonin may be helpful for sleep regulation if this is contributing to agitation.

#### Agitation Due to Substance Intoxication or Withdrawal

In cases of known or suspected substance intoxication or withdrawal, medication choice should be dictated by clinical presentation and the suspected substances. If urine toxicology is indicated, and the results are negative, newer synthetic drugs, such as synthetic cannabinoids and cathinones, should be suspected. Gas chromatography and mass spectrometry, where available, can also help to identify an ingestion. If the substance ingested is unknown, there is consensus that lorazepam should be used, and potentially combined with haloperidol if the patient is severely agitated or hallucinating.

#### Agitation in a Patient with Developmental Delay or Autism Spectrum Disorder

Youth with autism or developmental disabilities can be particularly vulnerable to ADEs from many of the medications commonly used to treat acute agitation, including benzodiazepines. Therefore, behavioral strategies are especially important in this population. Youth with autism or developmental disabilities often become agitated in the context of unrecognized physical or sensory discomfort, including headache, dental pain, gastrointestinal distress/constipation, and overstimulation. A detailed history from parents or guardians and close observation/examination can often elucidate potential triggers and inform treatment. A care plan with a list of specific triggers and calming strategies helps coordinate care across shifts in the ED setting. Asking parents or guardians about the child’s prior medication responses, either positive or negative, can also inform choice of PRN medication. An extra dose of the child’s regular standing medication may be preferable given risk of ADEs. IM administration should be avoided unless absolutely necessary for safety.

#### Agitation in the Context of Acute Psychiatric Illness

Agitation can occur in youth with a range of psychiatric illnesses, both acute and chronic. Missing home medications, at times due to waiting in the ED, is a frequent cause of agitation, so administering those home medications or administering an extra half or full dose can be effective. Youth with chronic psychiatric illness may alternatively become agitated for reasons that have nothing to do with their illness (e.g., a teen with a history of bipolar mania who is delirious, intoxicated, or in severe pain). Clinicians should also recall that mania and psychosis are rare in preadolescents; thus, a child presenting with agitation with disorganized thinking/behavior, hallucinations or delusions is more likely to be delirious, catatonic, or having difficulty communicating his or her experiences due to autism, intellectual disability, or psychological trauma.

#### Agitation of Unknown Etiology

While every effort should be made to identify the etiology of agitation, there will be patients for whom this is not possible, and the clinician should use his or her best judgment. For mild agitation, de-escalation strategies should be used while triggers for agitation are assessed. For moderate agitation, lorazepam, diphenhydramine or olanzapine can be used (though olanzapine and lorazepam should not be co-administered). For severe agitation, lorazepam can be combined with haloperidol, or chlorpromazine, or olanzapine can be used as single agents.

## DISCUSSION

While there was consensus as to general principals of medication use for agitation and some specific agents and strategies as described above, there was not consensus to support the use of one medication or even class of medication for all patients. This reflects both the absence of a strong evidence base, heterogeneity of the patient population, multifactorial nature of agitation, and practice differences between hospitals, regions, training programs, and individuals.

The specific ED setting will also have significant influence on choice of medication for agitation, and even on when medications are indicated. In the situation of an unlocked medical ED containing numerous pieces of equipment with which a child could (purposefully or accidentally) harm himself or herself or others, it may be faster to medicate an agitated child than in a psychiatric ED with specialized staff and an environment designed for safety. Psychiatric EDs, however, rarely have child life support that can be crucial in preventing agitation among young or developmentally-delayed children in a pediatrics ED. Medical or pediatric EDs can administer IV medications compared to psychiatric EDs, which typically use IM medications if PO is not possible. Medical EDs may be more comfortable with potential ADEs such as QT prolongation or respiratory suppression if they have rapid or routine access to telemetry or airway support, but may balk at using unfamiliar psychiatric medications like chlorpromazine. Psychiatric EDs often lack immediate access to pediatric or emergency medicine support, which may complicate assessment and management of delirium or catatonia secondary to physical illness. Hospital formulary, tradition, and milieu preferences will also influence medication choice.

While these consensus guidelines are written largely with psychiatrists and child psychiatrists in mind, they are informed by expert consensus from providers with training in pediatrics and consultation psychiatry. We anticipate these guidelines may also be of use to pediatricians and EPs working in ED settings without immediate access to psychiatry consultation. When available, psychiatric consultants can help elucidate the etiology of agitation. Psychiatric consultation can also assist with the choice of medication and ongoing non-pharmacologic de-escalation strategies. Especially if a first dose of medication for an agitated child was not effective, psychiatry should be consulted to reevaluate the differential diagnosis and the pharmacologic and non-pharmacologic treatment plan. Psychiatry consultation should also be obtained for patients with more complex psychiatric pathology and those who are on complex regimens already, patients with a history of paradoxical reaction to medication, and patients with agitation of mixed etiology. Involvement of other mental health providers, including psychologists and social work, can be helpful in the diagnostic assessment as well as implementation of non-pharmacologic management strategies.

## LIMITATIONS

This report describes the results of expert consensus guidelines for psychopharmacologic management of agitation among pediatric patients in the ED. These guidelines are based on a systematic review of the literature, a review of existing guidelines and hospital protocols, and utilization of an accepted and evidence-based, consensus generation process designed to reduce bias. However, these guidelines are still predominantly based on expert opinion. They have not been tested for efficacy either in isolation or in comparison to existing guidelines or hospital protocols.

## CONCLUSION

In summary, while agitation in the ED occurs frequently and with high costs to patients and clinical programs, there is vastly insufficient research into the understanding, prevention, assessment or treatment of agitation in this context. Further research is needed in many areas of pediatric emergency psychiatry, and especially into the comparative efficacy of different medications for agitation in different types of patients, and into the efficacy of these medications compared to placebo or to non-pharmacologic de-escalation strategies.^28^–^29^ ED nursing and staff, pediatricians, emergency physicians, and adult psychiatrists need training in rapid diagnosis and stabilization of agitated youth, as well as support for non-pharmacologic de-escalation and crisis management. Computerized/electronic medical record-based assessment and risk stratification tools may be useful, as may be clinical pathways directed at providing support and ancillary services (child life, psychiatric, or social work consult) to at-risk youth before agitation occurs.

## Figures and Tables

**Figure:**
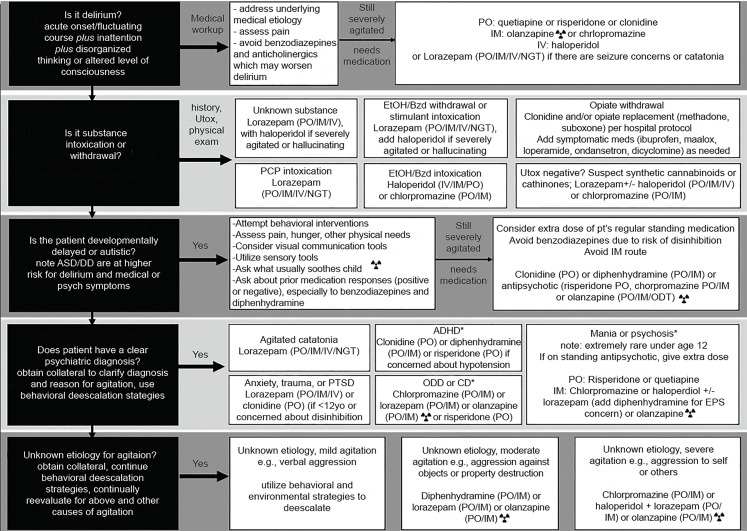
Clinical decision flow chart. *PO*, by mouth; *IM*, intramuscular; *IV*, intravenous; *NGT*, nasogastric tube; *EtOH*, ethanol; *Utox*, urine toxicology; *PCP*, phencyclidine; *EPS*, extrapyramidal symptoms; *ASD*, autism spectrum disorder; *DD*, developmental disability; *BZD*, benzodiazepines; *ODT*, orally dissolving tablet; *PTSD*, post-traumatic stress disorder; *ADHD*, attention deficit hyperactivity disorder; *ODD*, oppositional defiant disorder; *CD*, conduct disorder; *pt*, patient. *For these etiologies, in absence of consensus, medication options are listed alphabetically; 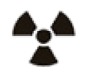 Do not give olanzapine and benzodiazepines within one hour of each other.

**Table 1:** Considerations when selecting a psychotropic for acute agitation management.

Medication factors
Formulas available
Onset and duration of action
Presence or absence of active metabolites
Interactions with other medications the patient has received in the ED or takes at home
Metabolism and exrcetion
Potential side effects or other drug effects that may be advantageous
Patient factors
Etiology or etiologies of agitation
Routes of administration available (PO, IV, IM, NGT)
GI function
Nutritional status and physical size
Hepatic function
Renal function
Other co-morbid physical health concerns
Desired response or effect on patient
Previous experience with psychotropics
Response to non-pharmacologic de-escalation strategies
Patient preference
Family expectation and family preference
System factors
Training and experience with non-pharmacologic approaches to agitation management and with use of different medications for agitation
Comfort of other work providers with use, monitorind and management of a given medication
Availability of monitoring practices within the care setting and hospital system

*ED,* emergency department; *PO*, by mouth; *IV*, intravenous; *GI*, gastrointestinal; *IM*; intramuscular; *NGT*, nasogastric tube.

**Table 2:** Medication reference.

Medication	Dose	Peak effect	Max daily dose	Notes/monitoring
Diphenhydramine (antihistaminic)	PO/IM: 12.5–50mg 1 mg/kg/dose	PO: 2 hours	Child: 50–100 mg Adolescent: 100–200 mg	Avoid in delirium. Can be combined with haloperidol or chlorpromazine if concerns for EPS. Can cause disinhibition or delirium in younger or DD youth.
Lorazepam (benzodiazepine)	PO/IM/IV/NGT: 0.5 mg-2 mg 0.05 mg-0.1 mg/kg/dose	IV: 10 minutes PO/IM: 1–2 hours	Child: 4 mg Adolescent: 6–8 mg Depending on weight/proir medication exposure	Can cause disinhibition or delirium in younger or DD youth. Can be given with haloperidol, chlorpromazine or risperidone. Do not give with olanzapine (especially IM due to risk of respiratory suppression.
Clonidine (alpha2 agonist)	PO: 0.05 mg-0.1 mg	PO: 30–60 minutes	27–40.5 kg: 0.2 mg/day 40.5–45 kg: 0.3 mg/day >45 kg: 0.4mg/day	Monitor for hypotension and bradycardia. Avaoid giving with BZD or atypicals due to hypotension risk.
Chlorpromazine (antipsychotic)	PO/IM: 12.5–60 mg (IM should be half PO dose) 0.55 mg/kg/dose	PO: 30–60 minutes IM: 15 minutes	Child <5 years: 40mg/day Child >5 years: 75mg/day	Monitor hypotension. Monitor for QT prolongation.
Haloperidol (antipsychotic)	PO/IM: 0.5 mg-5 mg (IM should be half a dose of PO) 0.55 mg/kg/dose	PO: 2 hours IM: 20 minutes	15–40 kg: 6mg >40 kg: 15 mg Depending on prior antipsychotic exposure	Monitor hypotension. Consider EKG or cardiac monitoring for QT prolongation, especially for IV administration. Note EPS risk with MDD > 3mg/day, with IV dosing having very high EPS risk. Consider AIMS testing.
Olanzapine (antipsychotic)	PO/ODT or IM: 2.5–10 mg (IM should be half or 1/4 dose of PO)	PO: 5 hours (range 1–8 hours) IM: 15–45 minutes	10–20 mg Depending on antipsychotic exposure	Do not give with or within 1 hour of any BZD given risk for respiratory suppresion
Risperidone (antipsychotic)	PO/ODT: 0.25–1mg 0.005–0.01mg/kg/dose	PO: 1 hour	Child: 1–2 mg Adolescent: 2–3 mg Depending on antipsychotic exposure	Can cause akathisia (restlessness/agitaion) in higher doses.
Quetiapine (antipsychotic)	PO: 25–50 mg 1–1.5 mg/kg/dose (or divided)	PO: 30 minutes-2 hours	>10 years: 600 mg Depending on prior antipsychotic exposure	More sedating at lower doses Monitor hypotension.

*PO,* by mouth; *IM*, intramuscular; *IV*, intravenous; *NGT*, nasogastric tube; *mg*, milligram; *EPS*, extrapyramidal symptoms; *DD*, developmental disability; mg/kg, milligrams per kilogram; *BZD*, benzodiazepines; *EKG*, electrocardiogram; *AIMS*, Abnormal Involuntary Movement Scale; *MDD*, major depressive disorder; *ODT*, orally dissolving tablet.
