# This Article Corrects: “Best Practices for Evaluation and Treatment of Agitated Children and Adolescents (BETA) in the Emergency Department: Consensus Statement of the American Association for Emergency Psychiatry”

**DOI:** 10.5811/westjem.2019.4.43550

**Published:** 2019-02-19

**Authors:** Ruth Gerson, Nasuh Malas, Vera Feuer, Gabrielle H. Silver, Raghuram Prasad, Megan M. Mroczkowski

**Affiliations:** * Bellevue Hospital/New York University, Department of Psychiatry, New York, New York; † University of Michigan, Departments of Psychiatry and Pediatrics, Ann Arbor, Michigan; ‡ Northwell Health, Department of Psychiatry, New Hyde Park, New York; § Weill Cornell Medical College, Department of Psychiatry, New York, New York; ¶ Children’s Hospital of Philadelphia, Department of Psychiatry, Philadelphia, Pennsylvania; || Columbia University Medical Center, Department of Psychiatry, New York, New York

*West J Emerg Med*. 2019 March;20(2):409–419

Best Practices for Evaluation and Treatment of Agitated Children and Adolescents (BETA) in the Emergency Department: Consensus Statement of the American Association for Emergency Psychiatry

Gerson R, Malas N, Feuer V, Silver GH, Prasad R, Mroczkowski MM

Erratum in

*West J Emerg Med*. 2019 May;20(3):537. Pediatric BETA Consensus Guideline Working Group members should not be included in full author list. The author list has now been corrected on this erratum.

Abstract

**Introduction:** Agitation in children and adolescents in the emergency department (ED) can be dangerous and distressing for patients, family and staff. We present consensus guidelines for management of agitation among pediatric patients in the ED, including non-pharmacologic methods and the use of immediate and as-needed medications.

**Methods:** Using the Delphi method of consensus, a workgroup comprised of 17 experts in emergency child and adolescent psychiatry and psychopharmacology from the the American Association for Emergency Psychiatry and the American Academy of Child and Adolescent Psychiatry Emergency Child Psychiatry Committee sought to create consensus guidelines for the management of acute agitation in children and adolescents in the ED.

**Results:** Consensus found that there should be a multimodal approach to managing agitation in the ED, and that etiology of agitation should drive choice of treatment. We describe general and specific recommendations for medication use.

**Conclusion:** These guidelines describing child and adolescent psychiatry expert consensus for the management of agitation in the ED may be of use to pediatricians and emergency physicians who are without immediate access to psychiatry consultation.

PMCID: PMC6404720 [PubMed - indexed for MEDLINE]

